# Utilizing micro-RNA-371a-3p for recurrence prediction in clinical stage I testicular cancerx

**DOI:** 10.1093/oncolo/oyag267

**Published:** 2026-07-11

**Authors:** Richard S Matulewicz, Andrea Knezevic, Adam Levin, Fei Ye, Darren R Feldman, Samuel A Funt

**Affiliations:** Department of Surgery, Memorial Sloan Kettering Cancer Center, New York, NY 10065, United States; Department Epidemiology and Biostatistics, Memorial Sloan Kettering Cancer Center, New York, NY 10065, United States; Department of Medicine, Memorial Sloan Kettering Cancer Center, New York, NY 10065, United States; Department of Pathology and Laboratory Medicine, Memorial Sloan Kettering Cancer Center, New York, NY 10065, United States; Department of Medicine, Memorial Sloan Kettering Cancer Center, New York, NY 10065, United States; Department of Medicine, Weill Cornell Medical College, New York, NY 10065, United States; Department of Medicine, Memorial Sloan Kettering Cancer Center, New York, NY 10065, United States; Department of Medicine, Weill Cornell Medical College, New York, NY 10065, United States

## Introduction

Testicular germ cell tumors (TGCTs), the most common cancer in adolescent and young adult men, are frequently diagnosed at clinical stage I (CSI) with active surveillance including serial cross-sectional imaging being the preferred management strategy.^1^^,^^2^ For the 15%-20% of CSI seminoma and 30% of CSI nonseminoma patients who develop metastatic disease, chemotherapy cures >90% of patients but has acute and, more importantly, chronic toxicities such as hearing loss and an increased risk of secondary cancers and heart disease.^2^^,^^3^ Early and accurate identification of patients with CSI TGCT at high or low risk for relapse after orchiectomy can inform adjuvant strategies that decrease the burden of treatment. Risk stratification efforts to date have focused on pathologic features of the primary tumor, but these approaches have suboptimal sensitivity and specificity. Thus, improved biomarkers are needed to better personalize management for patients with CSI TGCT and to avoid both under- and overtreatment.

MicroRNA-371a-3p (miR-371) is expressed by all non-teratomatous GCT components and can be measured in blood by reverse transcription-polymerase chain reaction. Multiple studies have demonstrated that circulating miR-371 expression has superior sensitivity and specificity in detecting disease than alpha-fetoprotein and human chorionic gonadotropin in patients with clinically active TGCT. Several groups have examined the performance of post-orchiectomy miR-371 in predicting relapse in patients with CSI TGCT. While two studies reported no association between miR-371 levels as a continuous variable and subsequent relapse,^4^^,^^5^ a third found that the miR-371 test interpreted as a dichotomous variable (positive vs negative) was strongly associated with relapse (hazard ratio [HR] 16.95; 95% confidence interval [CI]: 2.117-135.7; *P* < .001).^6^ However, these were retrospective series using archival samples procured in a unstandardized fashion, and investigators were not blinded.

## The CLIMATE (ANZUP 1906) study

At the 2026 Genitourinary Cancers Symposium of the American Society of Clinical Oncology, Tran et al.^7^ presented the interim results of the CLIMATE (ANZUP 1906) study, which was a prospective evaluation of the positive and negative predictive values (PPV and NPV) of a baseline, post-orchiectomy, dichotomous miR-371 result in predicting recurrence in CSI TGCT. Investigators were blinded to the miR-371 results, and blood samples were uniformly procured with predefined miR-371 testing methodology.

One hundred and ninety-six baseline plasma and serum samples were analyzed (60% seminoma and 40% nonseminoma) with a median time from orchiectomy to collection of 29 days (range 4-66 days) and a median patient follow-up of 18.9 months. There were 10 (8%) recurrences in patients with seminoma at a median of 6.7 months (vs expected 14 months^8^) and 30 (38%) recurrences in patients with nonseminoma at a median of 4.0 months (vs expected 5 months^9^). Similar to a prior report,^10^ the investigators found good qualitative concordance between plasma and serum results of 89%. Because plasma outperformed serum in predicting relapse (area under the curve 0.77 vs 0.69; *P* = .04), the investigators subsequently focused on the plasma results.

The risk of recurrence was significantly increased if the baseline plasma miR-371 result was positive compared with negative (HR 10.3; 95% CI, 5.3-19.8; *P* < .001) with 2-year recurrence free survival rates of 32.4% versus 88.9%, respectively. Sensitivity was 65% and specificity 90%. The NPV was 91% with 14 false negatives and PPV was 62% with 16 false positives. An exploratory analysis restricting all non-relapsing patients to >2 years follow-up and revealing an NPV of 75% and PPV of 93% suggests that the NPV may decrease and PPV may increase with additional follow-up. Importantly, when evaluating within histologic groups (seminoma or nonseminoma) among patients classified as intermediate or high risk for relapse based on features in the primary tumor,^11^^,^^12^ miR-371 remained associated with relapse in intermediate/high-risk seminoma (HR 21.9; 95% CI, 2.5-189.0) and in nonseminoma with lymphovascular invasion (HR 4.2; 95% CI, 1.3-13.7).

## Future directions

Although the interim results from the prospective CLIMATE found that plasma miR-371 positivity was a strong predictor of relapse in CSI TGCT and performed better than existing stratification strategies, the existing data is insufficient to support widespread clinical implementation. SWOG 1823 **(**NCT04435756) and AGCT 1531 (NCT03067181) are ongoing studies for evaluating miR-371 in early-stage TGCT that will provide additional validation. In addition, miR-371 is not widely available as a commercial test in North America and is currently performed in academic institutions using variable methodology and inconsistent diagnostic thresholds. The “teratoma problem” also remains (teratoma does not express miR-371),^13^ though the risk of pure teratomatous relapse is rare in CSI NSGCT and should never happen with pure seminoma. Circulating tumor DNA (ctDNA) detection is widely commercially available but is significantly more expensive and time consuming than miR-371 measurement. Although early reports suggest its utility for TGCT detection and post-surgical risk stratification,^14^ further investigation is needed. Relapse patterns in patients with positive miR-371 from CLIMATE and other ongoing studies will provide valuable insights on the feasibility of retroperitoneal lymph node dissection (RPLND) as a curative option for these patients. Finally, the impact of introducing this new test on patient decision-making and anxiety levels also warrants careful evaluation.

While the existing data do not yet support the use of post-orchiectomy miRNA results in routine clinical practice, they do support the design of interventional trials to elucidate its role in informing adjuvant treatment and adjusted surveillance intensity. For example, the MAGESTIC (MiRNA in detecting Active GCT in Early Suspected and metastaTIC disease; NCT06060873) and CLARITY (Clinical Trial Assessing microRNA-based Selection for RPLND In Testicular Cancer; NCT ID pending) trials are miR-371 guided RPLND studies in CSII and CSI TGCTs, respectively, aimed at validating the accuracy of positive miR-371 in detecting active GCTs at the time of surgery. Further, studies are warranted to determine whether incorporation of miR-371 into surveillance paradigms for CSI TSGCT can decrease the dependence on repeated cross-sectional imaging. As data mature from interventional trials, we remain hopeful that clinical implementation of miR-371 will inform optimal management of early-stage TGCT, improving risk stratification and judicious application of adjuvant therapy, thereby minimizing unnecessary toxicity.

## Author contributions

Richard S. Matulewicz (Investigation, Methodology, Supervision, Writing—original draft, Writing—review & editing), Andrea Knezevic (Investigation, Writing—review & editing), Adam Levin (Investigation, Writing—review & editing), Fei Ye (Investigation, Writing—review & editing), and Darren R. Feldman (Investigation, Writing—review & editing), and Samuel Funt (Conceptualization, Funding acquisition, Investigation, Methodology, Supervision, Writing—original draft, Writing—review & editing)

## Funding

This work was supported by a Cancer Center Program Grant (P30 CA008748). R.S.M. is supported by a 2023 MSK Department of Surgery Faculty Research Award and a 2025 Chanel Foundation for Survivorship Research Grant. S.A.F. is supported by an American Cancer Society Research Scholars Grant (ACS RSG-22–105-01-CSCT).

## References

[1] Scott AR , Stoltzfus KC, Tchelebi LT, et al Trends in cancer incidence in US adolescents and young adults, 1973-2015. JAMA Netw Open. 2020;3:e2027738. 10.1001/jamanetworkopen.2020.27738 33258907 PMC7709088

[2] Singla N , Bagrodia A, Baraban E, Fankhauser CD, Ged YMA. Testicular germ cell tumors: a review. JAMA. 2025;333:793-803. 10.1001/jama.2024.27122 39899286

[3] Feldman DR , Bosl GJ, Sheinfeld J, Motzer RJ. Medical treatment of advanced testicular cancer. JAMA. 2008;299:672-684. 10.1001/jama.299.6.672 18270356

[4] Lobo J , Leão R, Gillis AJM, et al Utility of serum miR-371a-3p in predicting relapse on surveillance in patients with clinical stage I testicular germ cell cancer. Eur Urol Oncol. 2021;4:483-491. 10.1016/j.euo.2020.11.004 33288479

[5] Belge G , Dumlupinar C, Nestler T, et al Detection of recurrence through microRNA-371a-3p serum levels in a follow-up of stage I testicular germ cell tumors in the DRKS-00019223 study. Clin Cancer Res. 2024;30:404-412. 10.1158/1078-0432.CCR-23-0730 37967143 PMC10792362

[6] Nappi L , Saxena N, Pautasso S, et al Long term follow-up analysis of plasma miR371 expression to detect early relapse in patients with clinical stage I testicular germ cell tumors on surveillance. JCO. 2023;41:5006-5006. 10.1200/JCO.2023.41.16_suppl.5006

[7] Tran B , Lewin JH, O’Haire S, et al Initial results from CLIMATE, a prospective cohort study assessing the clinical utility of miR-371a-3p (miR-371) as a marker of minimal residual disease (MRD) in clinical stage 1 testicular germ cell tumour (TGCT): ANZUP 1906. J Clin Oncol. 2026;44:586-586. 10.1200/JCO.2026.44.7_suppl.58641337691

[8] Kollmannsberger C , Tandstad T, Bedard PL, et al Patterns of relapse in patients with clinical stage I testicular cancer managed with active surveillance. J Clin Oncol. 2015;33:51-57. 10.1200/JCO.2014.56.2116 25135991

[9] Daugaard G , Gundgaard MG, Mortensen MS, et al Surveillance for stage I nonseminoma testicular cancer: outcomes and long-term follow-up in a population-based cohort. J Clin Oncol Off J Am Soc Clin Oncol. 2014;32:3817-3823. 10.1200/JCO.2013.53.5831 25267754

[10] Baky F , Matulewicz RS, Knezevic A, et al Comparison of serum and plasma microRNA expression levels in patients with germ cell tumours. BJU Int. 2025;135:748-750. 10.1111/bju.16644 39789892 PMC13133686

[11] Daugaard G , Gundgaard MG, Mortensen MS, et al Surveillance for stage I nonseminoma testicular cancer: outcomes and long-term follow-up in a population-based cohort. J Clin Oncol. 2014;32:3817-3823. 10.1200/JCO.2013.53.5831 25267754

[12] Boormans JL , Sylvester R, Anson-Cartwright L, et al Prognostic factor risk groups for clinical stage I seminoma: an individual patient data analysis by the European Association of Urology testicular cancer guidelines panel and guidelines office. Eur Urol Oncol. 2024;7:537-543. 10.1016/j.euo.2023.10.014 37951820

[13] Yodkhunnatham N , Pandit K, Puri D, Yuen KL, Bagrodia A. MicroRNAs in testicular germ cell tumors: the teratoma challenge. Int J Mol Sci. 2024;25:2156. 10.3390/ijms25042156 38396829 PMC10889716

[14] Ben-David R , Eraky A, Hug B, et al Clinical utility of tumour-informed circulating tumour DNA in patients with testicular cancer. BJU Int. 2025;136:1098-1106. Published online October 7, 10.1111/bju.16926 41054348

